# Ethyl 4-chloro-3-nitro­benzoate

**DOI:** 10.1107/S1600536807068304

**Published:** 2008-01-25

**Authors:** Hao-Yuan Li, Bo-Nian Liu, Shi-Gui Tang, Ye-Ming Xu, Cheng Guo

**Affiliations:** a College of Science, Nanjing University of Technology, Xinmofan Road No. 5 Nanjing, Nanjing 210009, People’s Republic of China; b College of Life Sciences and Pharmaceutical Engineering, Nanjing University of Technology, Nanjing 210009, People’s Republic of China

## Abstract

In the mol­ecule of the title compound, C_9_H_8_ClNO_4_, an intra­molecular C—H⋯O hydrogen bond results in the formation of a planar five-membered ring, which is nearly coplanar with the adjacent six-membered ring, the rings being oriented at a dihedral angle of 4.40 (3)°. In the crystal structure, inter­molecular C—H⋯O hydrogen bonds link the mol­ecules.

## Related literature

For related literature, see: Jönsson *et al.* (2004 ▶). For bond-length data, see: Allen *et al.* (1987 ▶).
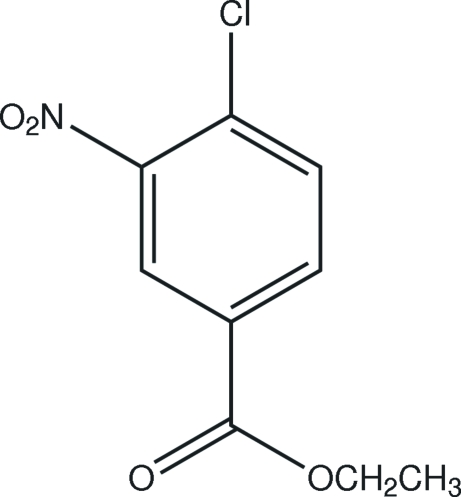


## Experimental

### 

#### Crystal data

C_9_H_8_ClNO_4_
*M* *_r_* = 229.61
Monoclinic, 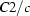

*a* = 12.930 (3) Å
*b* = 7.4820 (15) Å
*c* = 20.945 (4) Å
β = 92.11 (3)°
*V* = 2024.9 (7) Å^3^
*Z* = 8
Mo *K*α radiation
μ = 0.37 mm^−1^
*T* = 298 (2) K
0.40 × 0.30 × 0.10 mm

#### Data collection

Enraf–Nonius CAD-4 diffractometer
Absorption correction: ψ scan (North *et al.*, 1968 ▶) *T*_min_ = 0.866, *T*_max_ = 0.964
1984 measured reflections
1984 independent reflections
1449 reflections with *I* > 2σ(*I*)
3 standard reflections frequency: 120 min intensity decay: none

#### Refinement

*R*[*F* ^2^ > 2σ(*F* ^2^)] = 0.045
*wR*(*F* ^2^) = 0.130
*S* = 1.06
1984 reflections
136 parameters
H-atom parameters constrained
Δρ_max_ = 0.16 e Å^−3^
Δρ_min_ = −0.21 e Å^−3^

### 

Data collection: *CAD-4 Software* (Enraf–Nonius, 1989 ▶); cell refinement: *CAD-4 Software*; data reduction: *XCAD4* (Harms & Wocadlo, 1995 ▶); program(s) used to solve structure: *SHELXS97* (Sheldrick, 2008 ▶); program(s) used to refine structure: *SHELXL97* (Sheldrick, 2008 ▶); molecular graphics: *SHELXTL* (Siemens, 1996 ▶); software used to prepare material for publication: *SHELXTL*.

## Supplementary Material

Crystal structure: contains datablocks global, I. DOI: 10.1107/S1600536807068304/hk2403sup1.cif

Structure factors: contains datablocks I. DOI: 10.1107/S1600536807068304/hk2403Isup2.hkl

Additional supplementary materials:  crystallographic information; 3D view; checkCIF report

## Figures and Tables

**Table 1:** Hydrogen-bond geometry (Å, °)

*D*—H⋯*A*	*D*—H	H⋯*A*	*D*⋯*A*	*D*—H⋯*A*
C2—H2*B*⋯O2	0.97	2.29	2.706 (3)	104
C8—H8*A*⋯O2^i^	0.93	2.53	3.357 (3)	148

Symmetry code: (i) 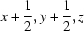.

**Fig. 1.**
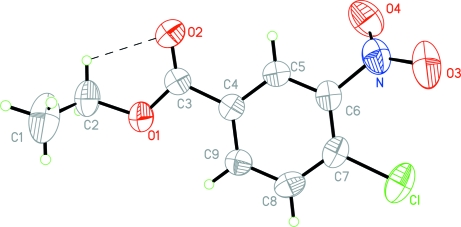
The molecular structure of the title molecule, with the atom-numbering scheme. Displacement ellipsoids are drawn at the 30% probability level.

**Fig. 2.**
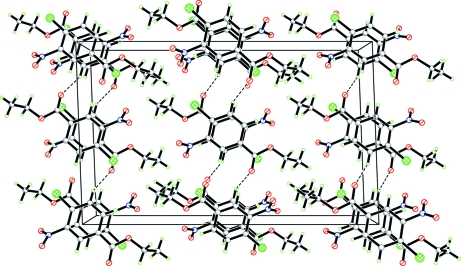
A packing diagram of (I). Hydrogen bonds are shown as dashed lines.

## supplementary crystallographic information

### Comment

Some derivatives of benzoic acid are important chemical materials. As part of our ongoing studies, we synthesized the title compound, (I), and report herein its crystal structure.

In the molecule of (I), (Fig. 1) the bond lengths and angles are within normal ranges (Allen *et al.*, 1987). The intramolecular C—H···O hydrogen bond (Table 1) results in the formation of a planar five-membered ring B (C2/H2B/C3/O1/O2). Ring A (C4—C9) is, of course, planar and the dihedral angle between them is A/B = 4.40 (3)°. So, rings A and B are also nearly co-planar.

In the crystal structure, intermolecular C—H···O hydrogen bonds (Table 1) link the molecules (Fig. 2), in which they may be effective in the stabilization of the structure.

### Experimental

For the preparation of the title compound, 4-chloro-3-nitrobenzoic acid (35.0 g, 174 mmol) was suspended in ethanol (150 ml) and cooled to 273 K. Concentrated sulfuric acid (15 ml) was slowly added with stirring, and then the mixture was heated under reflux for 17 h. Upon cooling to room temperature, a precipitate formed, which was collected by filtration and washed with cold ethanol (2 × 50 ml) and hexane (2 × 50 ml) to afford the ethyl ester as a white solid (yield; 29.9 g, 75%) (Daniel *et al.*, 2004). Crystals of (I) suitable for X-ray analysis were obtained by slow evaporation of a methanol solution.

### Refinement

H atoms were positioned geometrically, with C—H = 0.93, 0.97 and 0.96 Å for aromatic, methylene and methyl H, respectively, and constrained to ride on their parent atoms, with *U*_iso_(H) = *xU*_eq_(C), where *x* = 1.5 for methyl H, and *x* = 1.2 for all other H atoms.

### Crystal data

**Table:**

C_9_H_8_ClNO_4_	*F*_000_ = 944
*M**_r_* = 229.61	*D*_x_ = 1.506 Mg m^−^^3^
Monoclinic, *C*2/*c*	Mo *K*α radiation λ = 0.71073 Å
Hall symbol: -C 2yc	Cell parameters from 25 reflections
*a* = 12.930 (3) Å	θ = 9–14º
*b* = 7.4820 (15) Å	µ = 0.37 mm^−^^1^
*c* = 20.945 (4) Å	*T* = 298 (2) K
β = 92.11 (3)º	Block, colorless
*V* = 2024.9 (7) Å^3^	0.40 × 0.30 × 0.10 mm
*Z* = 8	

### Data collection

**Table:**

Enraf–Nonius CAD-4 diffractometer	*R*_int_ = 0.0000
Radiation source: fine-focus sealed tube	θ_max_ = 26.0º
Monochromator: graphite	θ_min_ = 2.0º
*T* = 298(2) K	*h* = −15→15
ω/2θ scans	*k* = 0→9
Absorption correction: ψ scan(North *et al.*, 1968)	*l* = 0→25
*T*_min_ = 0.866, *T*_max_ = 0.964	3 standard reflections
1984 measured reflections	every 120 min
1984 independent reflections	intensity decay: none
1449 reflections with *I* > 2σ(*I*)	

### Refinement

**Table:**

Refinement on *F*^2^	Secondary atom site location: difference Fourier map
Least-squares matrix: full	Hydrogen site location: inferred from neighbouring sites
*R*[*F*^2^ > 2σ(*F*^2^)] = 0.045	H-atom parameters constrained
*wR*(*F*^2^) = 0.130	*w* = 1/[σ^2^(*F*_o_^2^) + (0.06*P*)^2^ + 1.5*P*] where *P* = (*F*_o_^2^ + 2*F*_c_^2^)/3
*S* = 1.06	(Δ/σ)_max_ < 0.001
1984 reflections	Δρ_max_ = 0.16 e Å^−^^3^
136 parameters	Δρ_min_ = −0.21 e Å^−^^3^
Primary atom site location: structure-invariant direct methods	Extinction correction: none

### Special details

**Table:**

Geometry. All e.s.d.'s (except the e.s.d. in the dihedral angle between two l.s. planes) are estimated using the full covariance matrix. The cell e.s.d.'s are taken into account individually in the estimation of e.s.d.'s in distances, angles and torsion angles; correlations between e.s.d.'s in cell parameters are only used when they are defined by crystal symmetry. An approximate (isotropic) treatment of cell e.s.d.'s is used for estimating e.s.d.'s involving l.s. planes.
Refinement. Refinement of *F*^2^ against ALL reflections. The weighted *R*-factor *wR* and goodness of fit *S* are based on *F*^2^, conventional *R*-factors *R* are based on *F*, with *F* set to zero for negative *F*^2^. The threshold expression of *F*^2^ > σ(*F*^2^) is used only for calculating *R*-factors(gt) *etc*. and is not relevant to the choice of reflections for refinement. *R*-factors based on *F*^2^ are statistically about twice as large as those based on *F*, and *R*- factors based on ALL data will be even larger.

### Fractional atomic coordinates and isotropic or equivalent isotropic displacement parameters (Å^2^)

**Table:**

	*x*	*y*	*z*	*U*_iso_*/*U*_eq_	
Cl	1.15365 (6)	0.38995 (10)	0.39251 (4)	0.0722 (3)	
O1	0.91716 (14)	0.1218 (3)	0.65000 (8)	0.0663 (6)	
O2	0.78374 (14)	0.0870 (3)	0.58085 (9)	0.0627 (5)	
O3	0.9430 (2)	0.3802 (4)	0.33433 (11)	0.1031 (9)	
O4	0.87451 (19)	0.1219 (3)	0.35272 (10)	0.0825 (7)	
N	0.92750 (19)	0.2507 (4)	0.36829 (10)	0.0628 (6)	
C1	0.8393 (3)	0.2219 (5)	0.74482 (16)	0.0967 (12)	
H1A	0.7999	0.1862	0.7807	0.145*	
H1B	0.8023	0.3127	0.7210	0.145*	
H1C	0.9051	0.2681	0.7598	0.145*	
C2	0.8556 (3)	0.0672 (5)	0.70361 (13)	0.0801 (10)	
H2A	0.8912	−0.0264	0.7277	0.096*	
H2B	0.7894	0.0207	0.6879	0.096*	
C3	0.87120 (18)	0.1287 (3)	0.59279 (11)	0.0427 (5)	
C4	0.94310 (16)	0.1938 (3)	0.54379 (10)	0.0387 (5)	
C5	0.90841 (17)	0.1923 (3)	0.48080 (10)	0.0408 (5)	
H5A	0.8426	0.1492	0.4700	0.049*	
C6	0.97087 (18)	0.2546 (3)	0.43393 (11)	0.0444 (5)	
C7	1.06993 (18)	0.3170 (3)	0.44914 (11)	0.0459 (6)	
C8	1.10485 (18)	0.3167 (3)	0.51210 (12)	0.0489 (6)	
H8A	1.1711	0.3576	0.5229	0.059*	
C9	1.04200 (17)	0.2561 (3)	0.55916 (11)	0.0446 (5)	
H9A	1.0660	0.2569	0.6016	0.054*	

### Atomic displacement parameters (Å^2^)

**Table:**

	*U*^11^	*U*^22^	*U*^33^	*U*^12^	*U*^13^	*U*^23^
Cl	0.0766 (5)	0.0632 (5)	0.0794 (5)	0.0006 (4)	0.0387 (4)	0.0099 (4)
O1	0.0616 (11)	0.0952 (15)	0.0424 (10)	−0.0104 (10)	0.0052 (8)	0.0096 (9)
O2	0.0497 (10)	0.0774 (13)	0.0612 (11)	−0.0127 (9)	0.0065 (8)	0.0051 (9)
O3	0.130 (2)	0.116 (2)	0.0628 (13)	−0.0096 (17)	0.0003 (14)	0.0378 (14)
O4	0.1026 (17)	0.0833 (16)	0.0601 (12)	0.0024 (14)	−0.0146 (11)	−0.0164 (11)
N	0.0721 (15)	0.0704 (16)	0.0461 (12)	0.0122 (13)	0.0057 (11)	0.0025 (12)
C1	0.109 (3)	0.109 (3)	0.074 (2)	0.028 (2)	0.037 (2)	0.012 (2)
C2	0.088 (2)	0.107 (3)	0.0461 (15)	−0.011 (2)	0.0176 (14)	0.0144 (16)
C3	0.0456 (13)	0.0368 (12)	0.0455 (13)	0.0046 (10)	0.0015 (10)	−0.0005 (10)
C4	0.0422 (11)	0.0301 (11)	0.0441 (12)	0.0041 (9)	0.0044 (9)	−0.0015 (9)
C5	0.0397 (11)	0.0347 (11)	0.0480 (13)	0.0031 (9)	0.0012 (9)	−0.0023 (10)
C6	0.0526 (13)	0.0385 (12)	0.0424 (12)	0.0085 (10)	0.0053 (10)	0.0005 (10)
C7	0.0517 (13)	0.0340 (12)	0.0531 (14)	0.0057 (10)	0.0157 (11)	0.0025 (10)
C8	0.0412 (12)	0.0402 (13)	0.0657 (16)	−0.0015 (10)	0.0054 (11)	−0.0038 (11)
C9	0.0424 (12)	0.0443 (13)	0.0472 (13)	0.0021 (10)	0.0009 (10)	−0.0032 (10)

### Geometric parameters (Å, °)

**Table:**

Cl—C7	1.724 (2)	C2—H2B	0.9700
O1—C3	1.319 (3)	C3—C4	1.492 (3)
O1—C2	1.459 (3)	C4—C5	1.378 (3)
O2—C3	1.191 (3)	C4—C9	1.388 (3)
N—O3	1.222 (3)	C5—C6	1.375 (3)
N—O4	1.220 (3)	C5—H5A	0.9300
N—C6	1.466 (3)	C6—C7	1.389 (3)
C1—C2	1.463 (5)	C7—C8	1.378 (4)
C1—H1A	0.9600	C8—C9	1.377 (3)
C1—H1B	0.9600	C8—H8A	0.9300
C1—H1C	0.9600	C9—H9A	0.9300
C2—H2A	0.9700		
			
C3—O1—C2	118.0 (2)	C5—C4—C9	119.3 (2)
O4—N—O3	124.9 (3)	C5—C4—C3	117.84 (19)
O4—N—C6	117.3 (2)	C9—C4—C3	122.8 (2)
O3—N—C6	117.7 (3)	C6—C5—C4	120.1 (2)
C2—C1—H1A	109.5	C6—C5—H5A	119.9
C2—C1—H1B	109.5	C4—C5—H5A	119.9
H1A—C1—H1B	109.5	C5—C6—C7	120.7 (2)
C2—C1—H1C	109.5	C5—C6—N	116.6 (2)
H1A—C1—H1C	109.5	C7—C6—N	122.6 (2)
H1B—C1—H1C	109.5	C8—C7—C6	119.1 (2)
O1—C2—C1	109.1 (3)	C8—C7—Cl	117.85 (19)
O1—C2—H2A	109.9	C6—C7—Cl	123.07 (19)
C1—C2—H2A	109.9	C9—C8—C7	120.3 (2)
O1—C2—H2B	109.9	C9—C8—H8A	119.9
C1—C2—H2B	109.9	C7—C8—H8A	119.9
H2A—C2—H2B	108.3	C8—C9—C4	120.5 (2)
O2—C3—O1	125.0 (2)	C8—C9—H9A	119.8
O2—C3—C4	123.5 (2)	C4—C9—H9A	119.8
O1—C3—C4	111.5 (2)		
			
C3—O1—C2—C1	−109.8 (3)	C3—C4—C5—C6	178.6 (2)
C2—O1—C3—O2	−3.0 (4)	C5—C4—C9—C8	0.3 (3)
C2—O1—C3—C4	177.6 (2)	C3—C4—C9—C8	−179.2 (2)
O4—N—C6—C5	−38.6 (3)	C4—C5—C6—C7	1.0 (3)
O3—N—C6—C5	138.0 (3)	C4—C5—C6—N	−179.2 (2)
O4—N—C6—C7	141.2 (3)	C5—C6—C7—C8	−0.3 (3)
O3—N—C6—C7	−42.2 (3)	N—C6—C7—C8	179.8 (2)
O2—C3—C4—C5	−5.1 (3)	C5—C6—C7—Cl	177.82 (17)
O1—C3—C4—C5	174.2 (2)	N—C6—C7—Cl	−2.0 (3)
O2—C3—C4—C9	174.4 (2)	C6—C7—C8—C9	−0.3 (3)
O1—C3—C4—C9	−6.2 (3)	Cl—C7—C8—C9	−178.58 (18)
C9—C4—C5—C6	−1.0 (3)	C7—C8—C9—C4	0.3 (4)

### Hydrogen-bond geometry (Å, °)

**Table:**

*D*—H···*A*	*D*—H	H···*A*	*D*···*A*	*D*—H···*A*
C2—H2B···O2	0.97	2.29	2.706 (3)	104
C8—H8A···O2^i^	0.93	2.53	3.357 (3)	148

Symmetry codes: (i) *x*+1/2, *y*+1/2, *z*.
